# Complete Video-Assisted Thoracoscopic Surgery and Traditional Open Surgery for Elderly Patients With NSCLC

**DOI:** 10.3389/fsurg.2022.863273

**Published:** 2022-03-18

**Authors:** Yi Mao, Zhaojia Gao, Yajun Yin

**Affiliations:** Department of Thoracic Surgery, The Affiliated Changzhou No. 2 People's Hospital of Nanjing Medical University, Changzhou, China

**Keywords:** non-small cell lung cancer, complete video-assisted thoracoscopic surgery, traditional open surgery, cardiopulmonary function, lymph node dissection

## Abstract

**Objective:**

To observe the efficacy of complete video-assisted thoracoscopic surgery (CVATS) and traditional open surgery (TOS) in the treatment of elderly patients with non-small cell lung cancer (NSCLC) and their influence on cardiopulmonary function.

**Methods:**

A total of 120 elderly patients with primary NSCLC who were treated surgically in our hospital from January 2018 to January 2021 were selected and divided into the study group and the control group according to the different surgical procedures, 60 patients in each group. CVATS was used in the observation group and TOS in the control group. The surgical indexes and cardiopulmonary function indexes were observed and compared between the two groups. The serum C-reactive protein (CRP) level and visual analog scale's (VAS) score of the patients at different time points were detected. The incidence of postoperative complications was compared between the two groups.

**Results:**

The perioperative indexes such as operation time were significantly different between the two groups (*p* < 0.05), but the number of lymph node dissection was not significantly different (*p* > 0.05). The serum CRP level and VAS score of the observation group were significantly lower than those of the control group on the 1st, 3rd, and 7th postoperative days (*p* < 0.05). There were significant differences in cardiopulmonary function between the two groups on the 7th postoperative day (*p* < 0.05). The incidence of adverse reactions in the observation group was significantly lower than that in the control group (*p* > 0.05).

**Conclusion:**

CVATS is effective in the treatment of NSCLC. Compared with TOS therapy, CVATS has less damage to cardiopulmonary function and fewer complications, which is conducive to the rehabilitation of elderly patients. It is a safe and reliable scheme for the treatment of elderly patients with NSCLC.

## Introduction

Due to the popularity of low-dose chest CT physical examination in recent years, the detection rate of early lung cancer manifested as lung ground-glass nodule has greatly improved. At the same time, with the aging of the population and the serious pollution of the surrounding environment, the proportion of elderly patients is also increasing gradually (1, 2). Non-small cell lung cancer (NSCLC) is a common malignant tumor disease, which has no specific typical symptoms in the early stage, and is usually diagnosed in the middle or late stage. At this time, it has lost the best treatment opportunity for surgical resection. For some early patients, at this time, the tumor focus is small, and there is no sign of diffusion or metastasis. At this point, surgical resection is still the main treatment for patients. Pulmonary lobectomy is currently the most commonly used treatment for early-stage NSCLC in clinics. Most of the traditional treatments are traditional open surgery (TOS). However, due to the weak constitution and poor lung function of elderly patients, there are many complications after lobectomy, and the early mortality is high (3).

CVATS is suitable for biopsy of nodular masses in lung, resection of benign masses in lung and lobectomy, etc. Compared with the TOS, the CVATS has the advantages of clearer tissue display of surgical field tissue, stronger advantages of minimally invasive and more precise operation. Complete video-assisted thoracoscopic surgery (CVATS) is performed through a small thoracotomy incision and two or three small incisions. It does not need chest support or ribs cutting during the operation and has little influence on the structure of the chest and the function of respiratory muscle, thus reducing the trauma and promoting postoperative recovery (4, 5). However, the effect and the prognosis of lymph node dissection need further study. In this study, we compared the clinical efficacy of CVATS and TOS in the treatment of elderly patients with NSCLC and the impact on the cardiopulmonary function of the patients in order to explore whether CVATS can bring better surgical outcomes for the elderly patients.

## Data and Methods

### General Information

A total of 120 elderly patients with primary NSCLC who were treated surgically in our hospital from January 2018 to January 2021 were selected and divided into a study group and a control group according to the different surgical procedures, 60 patients in each group. CVATS was used in the observation group, and TOS was used in the control group. Inclusion criteria: all the patients were diagnosed with NSCLC by imaging and pathology before operation and had clinical stages of stage I-III a; all the patients can tolerate the surgical treatment and have good compliance after preoperative evaluation. Exclusion criteria: the patients with preoperative radiotherapy and chemotherapy or dysfunction of important organs, such as heart, liver and brain; exclusion of other types of lung tumors and distant metastasis; the patients with previous thoracic surgery were excluded.

### Research Methods

TOS was adopted in the control group. After tracheal intubation and general anesthesia, a 20–25-cm incision was made in the fifth intercostal space. After anatomical lobectomy, the lymph nodes were systematically cleaned. After the operation, a drainage tube was placed through the midline of the armpit of the seventh intercostal space.

CVATS was used in the observation group. After general anesthesia, the patient was placed in the healthy lateral position, and an incision of about 1.5–2 cm in length was made between the 7th rib at the midline level of armpit, which was inserted into thoracoscope. A surgical incision was made in the 4th or 5th intercostal space at the level of axillary front line as the main surgical incision, with the length of about 2–3 cm, and a surgical incision was made in the 7th intercostal space at the level of scapular downline as the auxiliary surgical incision with the length of about 1.5–2 cm. The operation of the surgical instruments was completely supervised by video-assisted thoracoscopy. The patient's thoracic adhesion, lesion site, tumor size, infiltration range, enlargement, and metastasis of mediastinal lymph nodes in thoracic cavity were investigated, and then lobectomy was performed. Sequentially dissect the hilar structure to continuously cut off the pulmonary vessels, bronchi, and pulmonary fissure, and then free the pulmonary veins and artery branches. Lung lobes were removed through the main operation hole, and the thoracic and mediastinal lymph nodes were cleaned systematically. After the surgery, the drainage tube was inserted through the axillary midline of the 7th intercostal space.

Both groups of the patients were given routine nursing and symptomatic treatment after the operation.

### Observation Indicators

(1) The operation indexes of the patients in the two groups, such as operation time, bleeding volume, number of lymph node cleanings, postoperative drainage volume, and hospital stay, were observed and compared.
(2) The peripheral venous blood samples of the patients in the two groups were collected preoperatively and on the 1st, 3rd, and 7th postoperative days, and the serum C-reactive protein (CRP) levels were detected and compared.
(3) The visual analog scale (VAS) was applied to evaluate and compare the pain severity on the 1st, 3rd, and 7th postoperative days. VAS scoring criteria: 0 point means no pain, 10 points mean the most pain, <3 points mean good analgesia, and ≥ 5 points mean poor analgesic effect.
(4) Perioperative cardiopulmonary indicators [heart rate (HR), forced expiratory volume in 1 s (FEV_1_), maximum voluntary ventilation (MVV), diffusion of lung CO (DLCO)] of the two groups were compared and analyzed.
(5) The incidence of postoperative complications was observed and compared between the two groups.

### Statistical Methods

SPSS22.0 software was used for processing. The measurement data of the experimental data were expressed as mean standard deviation ($\bar{x}$ ± s). All the data were in normal distribution, and the means between the two groups were compared by independent sample *t*-test. ANOVA was used to compare multiple time points. The count data were expressed as (%), and the comparison was performed using χ^2^ test. The test level was α = 0.05, and *p* < 0.05 indicated that the difference was statistically significant.

## Results

### Patients With General Data Comparison

There was no significant difference in general information, such as gender, age, disease type, pathological type, clinical stage, and differentiation degree between the two groups (*p* > 0.05), indicating that they were comparable, as shown in Table 1.

**Table 1:** Comparison of general data of patients between the two groups (*n*, $\bar{x}$ ± s).

**Group**	**Gender**		**Disease type**		**Pathological type**		
	**Male**	**Female**	**Central**	**Peripheral**	**Glandular**	**Squamous**	**Other**
			**type**	**type**	**cancer**	**carcinoma**	
Control group	37 (61.67)	23 (38.33)	31 (51.67)	29 (48.33)	22 (36.67)	35 (58.33)	3 (5.00)
(*n =* 60)
Observation group	35 (58.33)	25 (41.67)	33 (55.00)	27 (45.00)	20 (33.33)	36 (60.00)	4 (6.67)
(*n =* 60)
*χ^2^ value*	0.139		0.134		0.252		
*P* value	0.709		0.714		0.882		
**Group**	**Age (years)**	**Clinical stages**			**Differentiation degree**		
		**Stage I**	**Stage II**	**Stage IIIa**	**Poorly differentiated**	**Intermediate differentiation**	**Highly differentiated**
Control group	67.83 ± 2.14	29 (48.33)	21 (35.00)	10 (16.67)	12 (20.00)	39 (65.00)	9 (15.00)
(*n =* 60)
Observation group	68.18 ± 2.87	27 (45.00)	22 (36.67)	11 (18.33)	14 (23.33)	41 (68.33)	5 (8.33)
(*n =* 60)
*t/χ^2^ value*	0.757	0.142			1.347		
*P* value	0.450	0.931			0.510		

### Comparison of Surgical Indicators Between the Two Groups

The operation time, bleeding volume, drainage volume, and hospital stay of the patients in the observation group were lower than those in the control group (*p* < 0.05). There was no significant difference in the number of lymph node dissection between the two groups (*p* > 0.05), as shown in Table 2.

**Table 2:** Comparison of surgical indexes between the two groups (*n*, $\bar{x}$ ± s).

		**Operation**	**Bleeding**	**Number of lymph**	**Drainage**	**Hospital**
**Group**	**N**	**time (min)**	**volume (ml)**	**node dissection (units)**	**volume (ml)**	**stay (d)**
Control group	60	179.62 ± 31.25	372.91 ± 70.28	18.06 ± 4.12	2086.61 ± 427.63	12.36 ± 2.95
Observation group	60	120.48 ± 33.94	246.83 ± 69.82	18.53 ± 3.92	1394.52 ± 357.19	7.05 ± 1.83
*t value*		9.929	9.858	0.640	9.621	11.848
*P* value		<0.001	<0.001	0.523	<0.001	<0.001

### Comparison of CRP Levels Between the Two Groups

**Figure 1:**
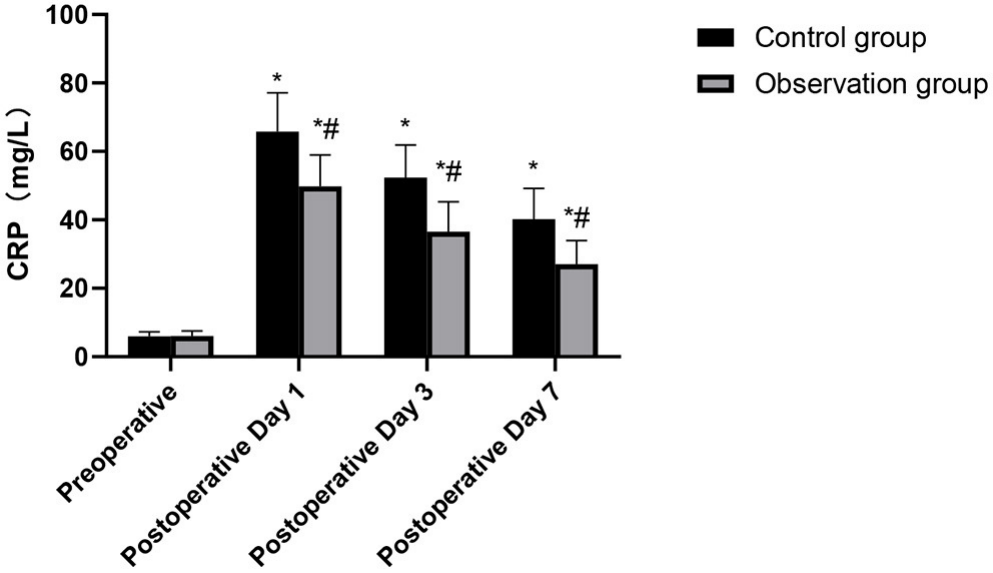
Comparison of CRP levels in the patients of the two groups. Compared with preoperative, *p < 0.05. Compared with the control group, ^#^*p* < 0.05.

Serum CRP levels preoperative between the two groups had no significant difference (*p* > 0.05), but it peaked on the 1st postoperative day and began to decline on the 3rd and 7th postoperative days. In addition, serum CRP levels of the patients in the observation group at each time point in postoperative were significantly lower than those in the control group (*p* < 0.05), as shown in Figure 1.

### Comparison of VAS Scores Postoperative Between the Two Groups

**Figure 2:**
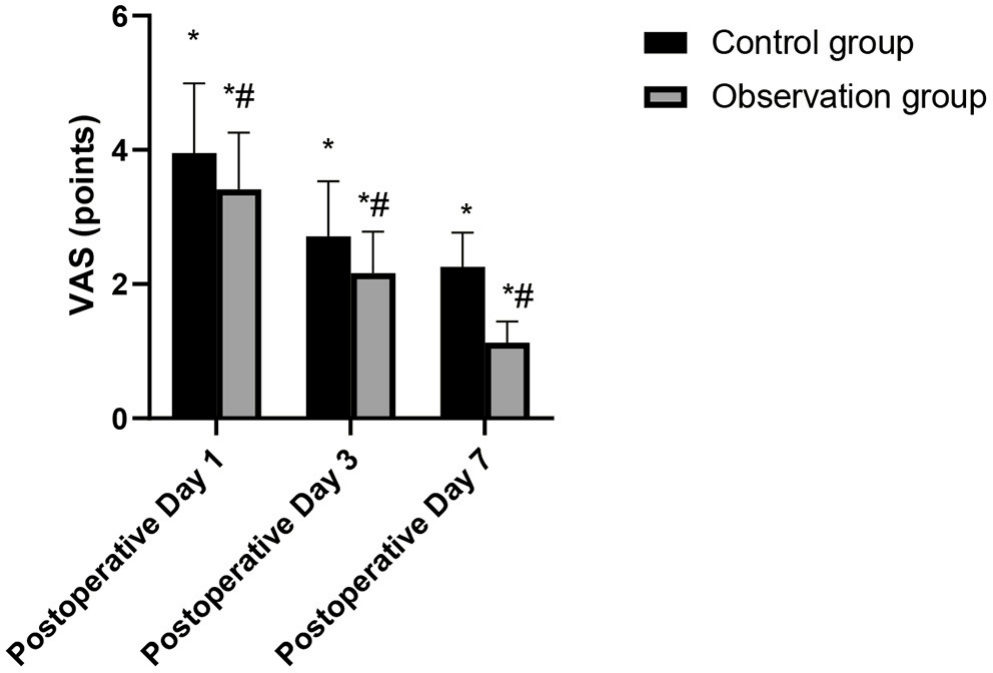
Comparison of VAS scores in the patients of the two groups. Compared with postoperative, *p < 0.05. Compared with the control group, ^#^*p* < 0.05.

The VAS scores of the patients in the two groups decreased sequentially at each time point postoperatively, and the VAS scores of the patients in the observation group at each time point at the 1st, 3rd, and 7th postoperative days were significantly lower than those in the control group (*p* < 0.05), as shown in Figure 2.

### Comparison of Cardiopulmonary Function Indexes During Postoperative Between the Two Groups

**Figure 3:**
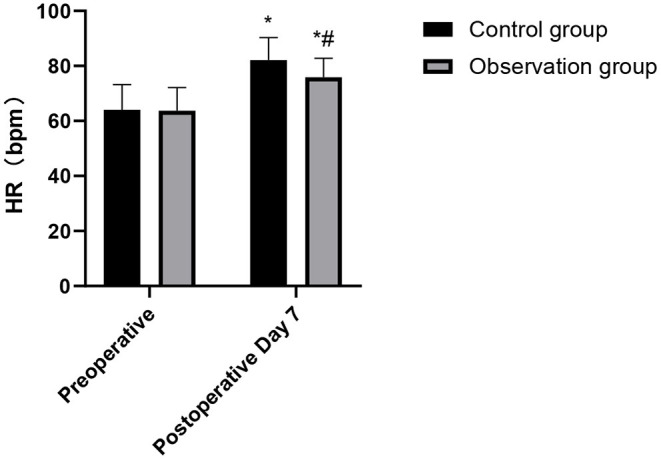
Comparison of HR changes in the patients of the two groups. Compared with preoperative, *p < 0.05. Compared with the control group, ^#^*p* < 0.05.

**Figure 4:**
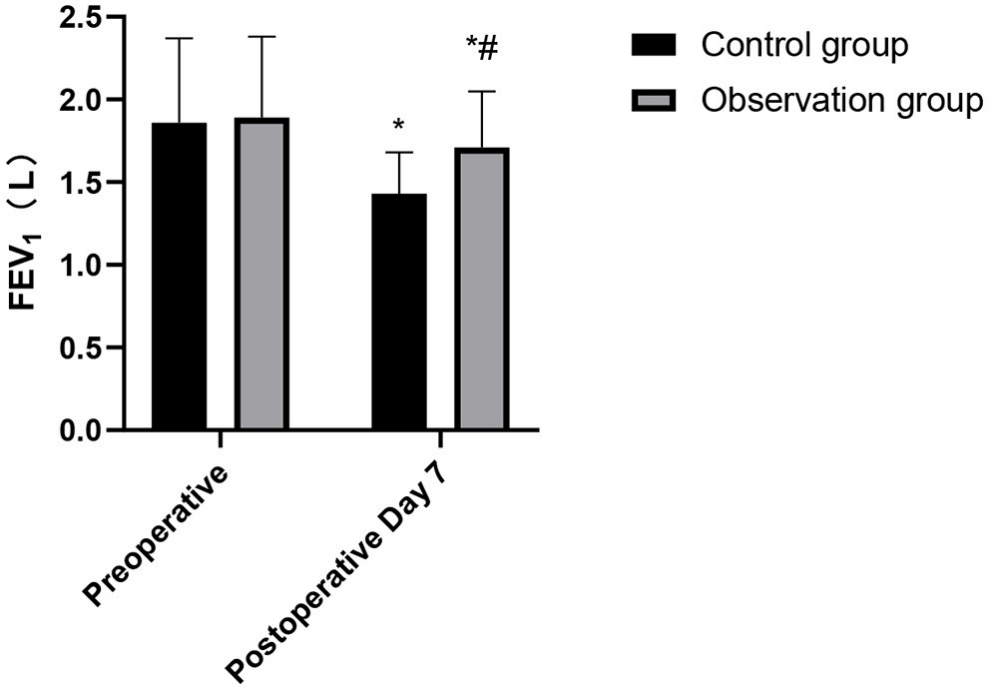
Comparison of FEV_1_ changes in the patients of the two groups. Compared with preoperative, *p < 0.05. Compared with the control group, ^#^*p* < 0.05.

**Figure 5:**
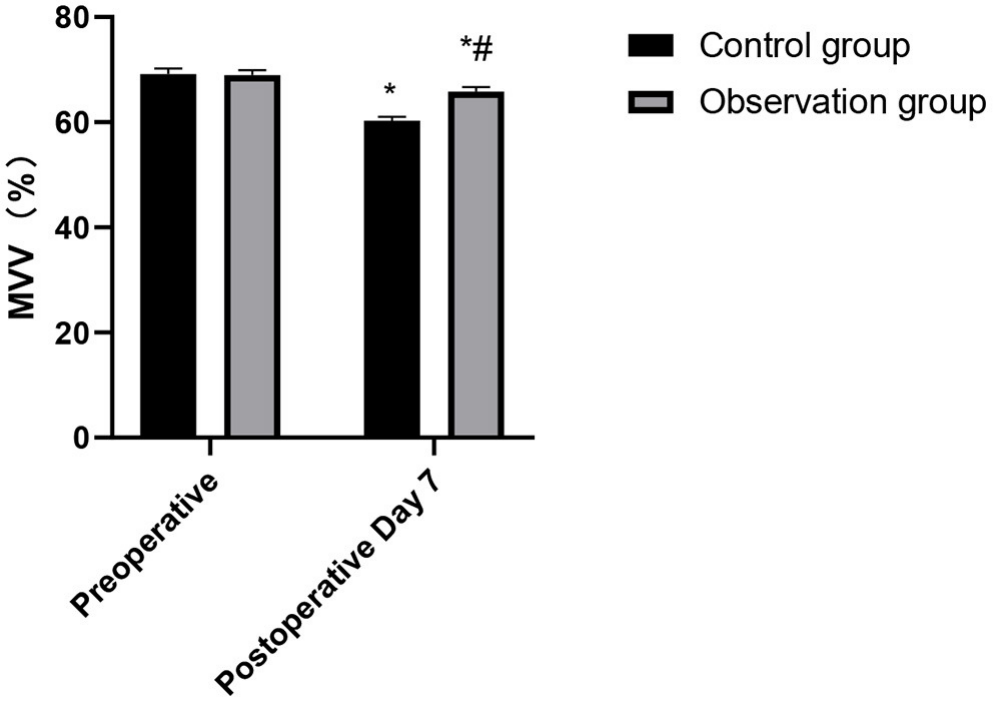
Comparison of MVV changes in the patients of the two groups. Compared with preoperative, *p < 0.05. Compared with the control group, ^#^*p* < 0.05.

**Figure 6:**
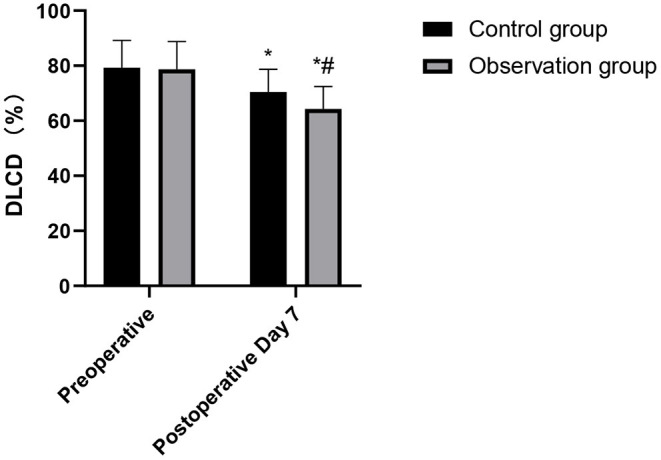
Comparison of DLCD changes in the patients of the two groups. Compared with preoperative, *p < 0.05. Compared with the control group, ^#^*p* < 0.05.

After the operation, the HR and DLCD in the observation group were higher than those in the control group, while FEV_1_ and MVV were lower than those in the control group. These differences were statistically significant (*p* < 0.05), as shown in Figures 3–6.

### Comparison of the Incidence of Adverse Reactions Between the Two Groups

**Figure 7:**
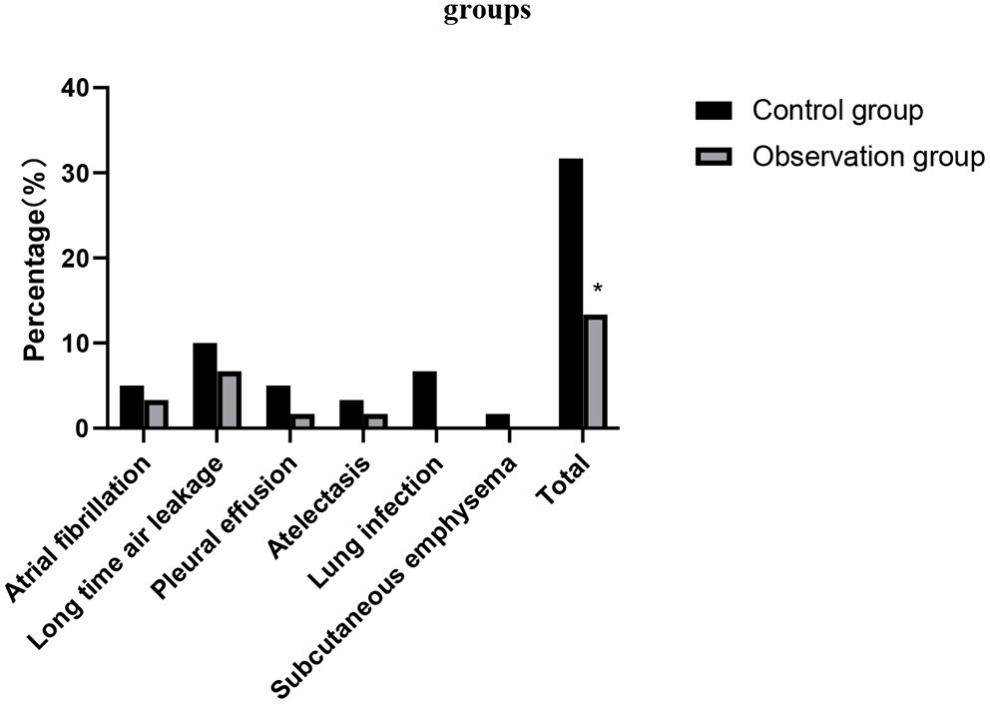
Comparison of the incidence of adverse reactions between the two groups. Compared with the control group, *p < 0.05.

The incidence of adverse reactions in the observation group was significantly lower than that in the control group (*p* > 0.05), as shown in Figure 7.

## Discussion

Because of the abundant blood supply to the lungs, blood metastasis can occur in the early stage, and there is no specific clinical manifestations. The main reason for poor treatment effect is the low early diagnosis rate (6). Although radiotherapy, chemotherapy, and biotherapy can prolong the survival time of patients with lung cancer, surgery is still the only way to achieve radical cure (7, 8). The objective of surgery is to remove as many lesions and lymph nodes as possible, and, at the same time, to maximize the preservation of intact lung tissue so as to ensure surgical effect and bring long-term survival benefits for patients. Elderly patients with lung cancer are a special group. Because of physiological factors and physical function degradation, young people are often diagnosed at an early stage, but they give up radical lobectomy because they cannot tolerate lobectomy. On the other hand, the elderly often have other diseases, which makes the treatment of elderly patients with lung cancer much more complicated (9, 10).

With the development of video-assisted thoracoscopic surgery and the skill of surgeons, it provides another option for elderly patients with lung cancer. Previous studies have shown that, compared with TOS, CVATS for patients with early NSCLC can relieve postoperative pain, have better postoperative lung function, shorter hospital stay, and a similar long-term survival rate (11, 12). CVATS has a clear vision and no blind spots, which is conducive to the accurate operation of doctors. To a greater extent, the accidental injuries during the surgery were reduced, and the amount of bleeding and postoperative drainage was further reduced. Besides, the surgical instruments are mature, accurate, and limited, which are convenient for lobectomy and lymph node dissection (13, 14). In this study, the perioperative indicators such as operation time were significantly different between the two groups of the patients, but the number of lymph node dissection was not significantly different. It is suggested that CVATS can not only achieve the lymph node dissection effect of traditional TOS but also can be safe and reliable, which accords with the principle of radical resection of tumor. CVATS requires a high degree of surgery, and the operator must be familiar with the skills of thoracic anatomy and endoscopy. The amount of bleeding and operation time are a comprehensive response of the operator to the degree of operation proficiency, and also a response of the operation to the degree of injury of the patient. The advantages of CVATS also lie in the fact that the surgical incision is small, and it is not necessary to cut off the intercostal muscles and ribs, so it has little effect on the muscular nerves and thoracic structure, which is conducive to reducing the degree of pain after surgery and has certain positive significance for the rehabilitation of patients after surgery (15, 16). This study showed that the VAS scores of the observation group at each time point postoperative were significantly lower than those in the control group.

At present, the comparison between CVATS and TOS is mostly based on some indexes such as clinical efficacy. In fact, in addition to the macroscopic indexes of clinical trauma, the evaluation of the effects of the two kinds of surgery can also be analyzed in the macroscopic aspect. In the case of infection or some tissue damage, CRP content usually increases rapidly, and it is a non-specific inflammatory marker (17, 18). In this study, the serum CRP levels in the observation group at each time point postoperative were significantly lower than those in the control group. The above results show that, compared with TOS, CVATS can reduce the patients' early injury, reduces the level of postoperative stress reaction, and relieves the body's inflammatory reaction, thus promoting the postoperative rehabilitation process (19).

Cardiopulmonary function of the human body can directly affect the activities of other organs and muscles. Cardiopulmonary function represents whether the patient's cardiopulmonary function is suitable for operation or not, and it is closely related to the occurrence of postoperative cardiopulmonary complications. If surgical trauma causes great damage to the cardiopulmonary function, the probability of postoperative cardiopulmonary complications will increase (20). It has been pointed out that CVATS is minimally invasive, and it has little influence on patients' immunity and cardiopulmonary function, thus contributing patients' rapid recovery (21). The results of this study showed that the cardiopulmonary function of the observation group was significantly better than that of the patients in the control group. TOS can damage thoracic integrity, causing damage to the intercostal nerves and great invasive damage to cardiopulmonary function in the early postoperative period. In contrast, CVATS does not need to divide muscles and ribs, and has less damage to the surrounding normal lung tissue, thus retaining the integrity of the patient's chest to the maximum extent. At the same time, CVATS can avoid the excessive injury to respiratory muscles (such as serratus anterior, latissimus dorsi, and intercostal muscles), which is of great clinical significance for the protection of postoperative cardiopulmonary function and is conducive to reducing the occurrence of postoperative cardiopulmonary complications (22, 23). Therefore, the cardiopulmonary function of the elderly patients using CVATS is better than TOS. The study also showed that the incidence of adverse reactions in the observation group was significantly lower than that in the control group. This shows that CVATS is safe for the elderly, and the incidence of complications can be controlled within an acceptable range (24, 25).

In summary, CVATS has a reliable curative effect on the treatment of NSCLC. Compared with TOS treatment, CVATS has less damage to cardiopulmonary function and fewer complications, which is conducive to the rehabilitation of elderly patients. CVATS is a safe and reliable scheme for the treatment of elderly patients with NSCLC. However, due to the small number of observations cases and short follow-up time, we still need to continue to explore in future studies to optimize the choice of surgical methods and realize the principle of optimal patient benefits.

## Data Availability Statement

The original contributions presented in the study are included in the article/supplementary material, further inquiries can be directed to the corresponding author/s.

## Ethics Statement

The studies involving human participants were reviewed and approved by the Ethics Committee of the Affiliated Changzhou No. 2 People's Hospital of Nanjing Medical University. The patients/participants provided their written informed consent to participate in this study.

## Author Contributions

YY was the supervisor of the entire study. All authors contributed to the article and approved the submitted version.

## Conflict of Interest

The authors declare that the research was conducted in the absence of any commercial or financial relationships that could be construed as a potential conflict of interest.

## Publisher's Note

All claims expressed in this article are solely those of the authors and do not necessarily represent those of their affiliated organizations, or those of the publisher, the editors and the reviewers. Any product that may be evaluated in this article, or claim that may be made by its manufacturer, is not guaranteed or endorsed by the publisher.

## References

[1] Duma N Santana-Davila R Molina JR. Non-small cell lung cancer: epidemiology, screening, diagnosis, and treatment. Mayo Clin Proc. (2019) 94:1623–40. 10.1016/j.mayocp.2019.01.013 31378236

[2] Alexander M Kim SY Cheng H. Update 2020: management of non-small cell lung cancer. Lung. (2020) 198:897–907. 10.1007/s00408-020-00407-5 33175991 PMC7656891

[3] Yang HX Woo KM Sima CS Bains MS Adusumilli PS Huang J. Long-term survival based on the surgical approach to lobectomy for clinical stage I nonsmall cell lung cancer: comparison of robotic, video-assisted thoracic surgery, and thoracotomy lobectomy. Ann Surg. (2017) 265:431–7. 10.1097/SLA.0000000000001708 28059973 PMC5033685

[4] Mun M Nakao M Matsuura Y Ichinose J Nakagawa K Okumura S. Video-assisted thoracoscopic surgery lobectomy for non-small cell lung cancer. Gen Thorac Cardiovasc Surg. (2018) 66:626–31. 10.1007/s11748-018-0979-x 30062622

[5] Tsou KC Hsu HH Tsai TM Chen KC Chen JS. Clinical outcome of subcentimeter non-small cell lung cancer after VATS resection: Single institute experience with 424 patients. J Formos Med Assoc. (2020) 119:399–405. 10.1016/j.jfma.2019.07.004 31375390

[6] Hung WT Cheng YJ Chen JS. Video-assisted thoracoscopic surgery lobectomy for lung cancer in nonintubated anesthesia. Thorac Surg Clin. (2020) 30:73–82. 10.1016/j.thorsurg.2019.09.002 31761286

[7] Ujiie H Gregor A Yasufuku K. Minimally invasive surgical approaches for lung cancer. Expert Rev Respir Med. (2019) 13:571–8. 10.1080/17476348.2019.1610399 31055977

[8] Pennathur A Brunelli A Criner GJ Keshavarz H Mazzone P Walsh G. Definition and assessment of high risk in patients considered for lobectomy for stage I non-small cell lung cancer: The American Association for Thoracic Surgery expert panel consensus document. J Thorac Cardiovasc Surg. (2021) 162:1605–18.e6. 10.1016/j.jtcvs.2021.07.030 34716030

[9] Jia J Guo B Yang Z Liu Y Ga L Xing G. Outcomes of local thoracic surgery in patients with stage IV non-small-cell lung cancer: a SEER-based analysis. Eur J Cancer. (2021) 144:326–40. 10.1016/j.ejca.2020.12.002 33388490

[10] Cao J Yuan P Wang Y Xu J Yuan X Wang Z. Survival rates after lobectomy, segmentectomy, and wedge resection for non-small cell lung cancer. Ann Thorac Surg. (2018) 105:1483–91. 10.1016/j.athoracsur.2018.01.032 29462591

[11] Batihan G Ceylan KC Usluer O Kaya SÖ. Video-assisted thoracoscopic surgery vs thoracotomy for non-small cell lung cancer greater than 5 cm: is VATS a feasible approach for large tumors? J Cardiothorac Surg. (2020) 15:261. 10.1186/s13019-020-01305-w 32948217 PMC7501690

[12] Ikeda N. Updates on minimally invasive surgery in non-small cell lung cancer. Curr Treat Options Oncol. (2019) 20:16. 10.1007/s11864-019-0614-9 30741372

[13] Drevet G Maury JM Ginoux M Tronc F. Morbi-mortalité de la résection pulmonaire pour cancer par chirurgie vidéo-assistée chez l'octogénaire [Short-term results of video-assisted lung cancer surgery in octogenarians]. Rev Mal Respir. (2020) 37:293–8. 10.1016/j.rmr.2020.01.007 32273117

[14] Tuminello S Liu B Wolf A Alpert N Taioli E Flores RM. Comparison of in-hospital and long-term outcomes of sublobar lung cancer surgery by VATS and open techniques. Am J Clin Oncol. (2018) 41:1149–53. 10.1097/COC.0000000000000440 29642076

[15] Raman V Yang CJ Deng JZ D'Amico TA. Surgical treatment for early stage non-small cell lung cancer. J Thorac Dis. (2018) 10:898–904. 10.21037/jtd.2018.01.172 29780636 PMC5945689

[16] Zhao R Shi Z Cheng S. Uniport video assisted thoracoscopic surgery (U-VATS) exhibits increased feasibility, non-inferior tolerance, and equal efficiency compared with multiport VATS and open thoracotomy in the elderly non-small cell lung cancer patients at early stage. Medicine (Baltimore). (2019) 98:16137. 10.1097/MD.0000000000016137 31305396 PMC6641850

[17] Motono N Iwai S Iijima Y Usuda K Uramoto H. Operative invasiveness does not affect the prognosis of patients with non-small cell lung cancer. BMC Pulm Med. (2020) 20:265. 10.1186/s12890-020-01264-x 33059654 PMC7558745

[18] Matsuoka K Yamada T Matsuoka T Nagai S Ueda M Miyamoto Y. Video-assisted thoracoscopic surgery for lung cancer after induction therapy. Asian Cardiovasc Thorac Ann. (2018) 26:608–14. 10.1177/0218492318804413 30249109

[19] Oda R Okuda K Osaga S Watanabe T Sakane T Tatematsu T. Long-term outcomes of video-assisted thoracoscopic surgery lobectomy vs. thoracotomy lobectomy for stage IA non-small cell lung cancer. Surg Today. (2019) 49:369–77. 10.1007/s00595-018-1746-4 30511319

[20] Gao Y Zhang H Li Y Wang D Ma Y Chen Q. Preoperative pulmonary function correlates with systemic inflammatory response and prognosis in patients with non-small cell lung cancer: results of a single-institution retrospective study. Oncotarget. (2017) 8:27489–501. 10.18632/oncotarget.14225 28039482 PMC5432351

[21] Bongiolatti S Gonfiotti A Vokrri E Borgianni S Crisci R Curcio C. Thoracoscopic lobectomy for non-small-cell lung cancer in patients with impaired pulmonary function: analysis from a national database. Interact Cardiovasc Thorac Surg. (2020) 30:803–11. 10.1093/icvts/ivaa044 32249900

[22] Zhang Y Gao Y. Effects of VATS lobectomy, VATS anatomic segmentectomy, and open thoracotomy on pulmonary function of patients with non-small cell lung cancer. Zhongguo Fei Ai Za Zhi. (2016) 19:700–4. 10.3779/j.issn.1009-3419.2016.10.15 27760602 PMC5973410

[23] Shi Q Diao Y Qian J. Application of single-hole thoracoscopic surgery combined with ERAS concept for respiratory function exercise in perioperative period of lung cancer. Zhongguo Fei Ai Za Zhi. (2020) 23:667–72. 10.3779/j.issn.1009-3419.2020.101.26 32434296 PMC7467983

[24] Watanabe A Miyajima M Mishina T Tsuruta K Takahashi Y Maki R. Video-assisted thoracoscopic surgery node dissection for lung cancer treatment. Surg Today. (2017) 47:1419–28. 10.1007/s00595-017-1494-x 28285463

[25] Deng HY Qiu XM Zhu DX Tang X Zhou Q. Video-assisted thoracoscopic sleeve lobectomy for centrally located non-small cell lung cancer: a meta-analysis. World J Surg. (2021) 45:897–906. 10.1007/s00268-020-05877-5 33230587

